# Type of Drain in Chronic Subdural Hematoma—A Systematic Review and Meta-Analysis

**DOI:** 10.3389/fneur.2020.00312

**Published:** 2020-04-22

**Authors:** Ladina Greuter, Nader Hejrati, Jehuda Soleman

**Affiliations:** ^1^Department of Neurosurgery, University Hospital of Basel, Basel, Switzerland; ^2^Faculty of Medicine, University of Basel, Basel, Switzerland

**Keywords:** chronic subdual hematoma (CSDH), subperiosteal drain, subdural drain, meta-analysis, recurrence (MeSH), systematic review

## Abstract

**Background:** Chronic subdural hematoma (cSDH) is one of the most common neurosurgical diseases, while burr-hole drainage is the most frequently used surgical treatment. Strong evidence exists that subdural drain (SDD) placement reduces recurrence rates. However, the insertion of a subperiosteal drain (SPD) was shown to lead to similar recurrence rates and less complications than SDD. The aim of this study is to provide a systematic review of the literature and conduct a meta-analysis of studies comparing SPD with SDD after burr-hole drainage of cSDH.

**Methods:** Pubmed and Embase databases were searched using a systematic search strategy to identify studies on drain location up to December 2019. Two independent researchers assessed the studies for inclusion and quality. Primary outcome measure was recurrence, while secondary outcome measures were drain misplacement, morbidity, mortality, and clinical outcome. Besides randomized controlled trials (RCT), we included non-randomized prospective cohort studies, as well as retrospective cohort studies. A fixed effects model was used if low heterogeneity (*I*^2^ < 50%) was present, otherwise a random effects model was used.

**Results:** Following removal of duplicates, we screened 1109 articles of which 10 articles were included in our qualitative and quantitative analyses. One study was an RCT, three were non-randomized prospective cohort studies, and the remaining articles were retrospective cohort studies or subgroup analysis. In these 10 articles, 1,553 patients were treated with SPD and 1782 patients with SDD. Comparing the recurrence rate of cSDH a significant difference was found between SPD and SDD insertion (11.9 and 12.3%; RR 0.8, 95% CI 0.67–0.97, *I*^2^ = 0%, z = −2.27, *p* = 0.02). SPD had significantly lower rates of drain misplacement and parenchymal injuries (1.2 and 7.8%; RR 0.17, 95% CI 0.07–0.42, *I*^2^ = 0%, z = −3.4, *p* = 0.0001), as well as morbidity (6.4 and 8.2%; RR 0.65, 95% CI 0.5–0.84, *I*^2^ = 44.5%, z = −3.32, *p* =0.0009). Mortality rates (5.0 and 4.6%; RR 0.83, 95% CI 0.6–1.14, *I*^2^ = 0%, z = −1.2, *p* = 0.25) and favorable clinical outcome (89.6 and 88.9%; RR 1.1, 95% CI 0.89–1.24, *I*^2^ = 54.2%, *t* = 0.98, *p* = 0.40) were comparable in both groups.

**Conclusion:** The insertion of SPD after burr-hole drainage of cSDH showed lower rates of recurrence, drain misplacements and parenchymal injuries, as well as overall morbidity, while clinical outcome and mortality were comparable to SDD. Therefore, the insertion of SPD after surgical drainage of cSDH should be encouraged.

## Background

Chronic subdural hematoma (cSDH) is one of the most common neurosurgical pathologies. CSDH can lead to substantial morbidity and mortality, which makes optimal treatment paramount (1). The gold standard of treatment remains surgical drainage of the hematoma through burr-hole trepanation and insertion of a drain (2). A randomized controlled trial (RCT) showed that the insertion of a subdural drain (SDD) after burr-hole drainage of cSDH significantly reduces the rate of recurrence and improves outcome (3). However, the insertion of SDD carries a risk of parenchymal injury due to their proximity to the cortex or bridging veins (4–7). Therefore, subperiosteal drains (SPD), which are inserted between the calvarium and the periosteum have been recommended by some surgeons (2, 6). Several studies, amongst others a recently published RCT, were carried out comparing these two different drain types, with regard to outcome and recurrence, with somewhat controversial results (4, 5, 7–14).

The aim of this systematic review and meta-analysis is to compare SPD and SDD with regard to rate of recurrence, morbidity, mortality, and clinical outcome.

## Methods

### Search Method and Data Analysis

**Figure 1:**
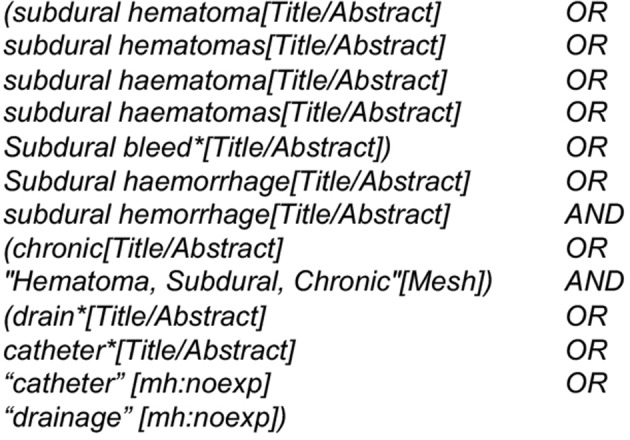
Detailed database search parameters used.

We used a search string with the keywords “chronic subdural hematoma” and “drain” in the databases Pubmed and Embase (Figure 1).

All results from Pubmed and Embase published until December 2019 were assessed by two of the authors independently (LG and NH). After removal of duplicates, all remaining articles were analyzed according to their titles. Abstracts were reviewed and a list of references was generated, while the remaining results underwent a full text evaluation and a final list of references was compiled. In case of disagreement concerning the inclusion of a study, the decision to include was made by a third researcher (JS).

### Inclusion Criteria and Outcome Measures

Besides randomized controlled trials (RCT), we included non-randomized prospective cohort studies, as well as retrospective cohort studies in our analysis. Technical reports, which described a novel drainage method but lacked to conduct a comparison between two different drainage types, as well as case reports or reviews were excluded from this report. The primary outcome measure was recurrence of cSDH, while secondary outcome measures were drain misplacement and intraparenchymal brain injury rate due to drain misplacement, overall morbidity (including drain misplacement, seizures, and infection), infection rate, mortality, and clinical outcome using modified Ranking Scale (mRS). We included only studies published in English.

Among the included studies, follow-up time points varied; hence, we combined the follow-up reports from 4–12 weeks postoperatively as we did not expect major clinical differences in this time period. Clinical outcomes assessed earlier than 1 month postoperatively were not included in our analysis. There was a heterogeneity of clinical outcome measures in the individual studies with either mRS or Glasgow Outcome Scale. For this analysis, we evaluated solely the mRS results, while favorable outcome was defined as mRS 0–3.

We defined the drains placed above the bone as “subperiosteal” drains rather than “subgaleal” drains. Some authors refer to these drains as subgaleal drains, however, in order to create burr-holes the periosteum needs to be scraped off the bone. Therefore, once the drain is then placed over the frontal and parietal burr-holes, it is automatically placed in a subperiosteal manner. The tunneling of the drain between the two burr-holes can be done subperiosteally or subgaleally, however the technique of tunneling is not essential for the drainage of the hematoma through the drain, but rather where the drains lay above the burr-holes. Therefore, in our opinion, the correct term is “subperiosteal” drain. To note, that not all authors describe the exact method of drain placement, therefore, theoretically some included studies might have used subgaleal drains.

### Quality Assessment

Risk of bias of RCTs was assessed by using the revised risk of bias (RoB-2) tool (15). Quality assessment of the non-randomized prospective cohort studies and retrospective cohort studies was carried out using Robins-1, respectively Newcastle Ottawa Scale (16, 17). Quality assessment was carried out individually and thereafter compared by two of the authors (LG, NH).

### Statistical Analysis

Risk ratio (RR) was used as an effect measure for the pooled outcomes. In case of low heterogeneity (*I*^2^ < 50%) the fixed-effects method was applied, otherwise the random-effects analysis was used. For the primary outcome measure, the so-called “leave one out” method was carried out, as an additional influence analysis. The results of the meta-analysis were recalculated K-1 times by sequentially leaving one study out to detect the studies which influence the overall effect the most. Finally, forest and funnel plots were generated and are presented for all outcomes.

All analyses were done using the R statistical software (version 3.6.2, 2019) with the help of the dmetar package (18). The review was performed in accordance with the Preferred Reporting Items for Systematic Reviews and Meta-Analyses (PRISMA) guidelines.

## Results

**Figure 2:**
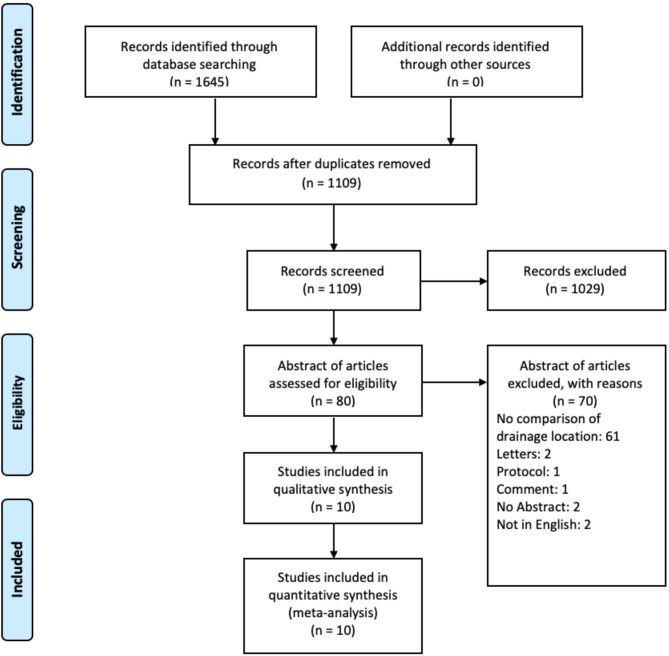
Flow Chart according to the PRISMA guidelines showing study selection.

After screening 1,109 articles, 10 were included in our qualitative systematic review as well as quantitative meta-analysis (Figure 2). One study was an RCT (7), three studies were non-randomized prospective cohort studies (4, 10, 12), and six were retrospective cohort studies or sub-group analysis of other studies (Table 1) (5, 8, 9, 11, 13, 14). For our analysis 1,553 patients from the SPD group and 1,782 patients from the SDD group were included.

**Table 1:** Overview of the included studies showing the number of patients within the subperiosteal drain (SPD) and subdural drain (SDD) group, as well as the primary outcome assessed for each study.

**References**	**Type of study**	**Primary outcome**	**Number of SPD**	**Number of SDD**
Häni et al. (8)	*post-hoc* subgroup analysis of single-center RCT	Recurrence	214	135
Zhang et al. (9)	Retrospective cohort study, multicenter	Recurrence, outcome	241	329
Soleman et al. (7)	RCT, multicenter, not blinded	Recurrence	120	100
Glancz et al. (11)	Subgroup analysis of multicenter prospective cohort study	Outcome	44	533
Ishfaq (10)	Prospective, non-randomized trial	Outcome	31	31
Sjavik et al. (13)	Multicenter retrospective comparative cohort study	Recurrence	764	496
Chih et al. (4)	Prospective, non-randomized trial, multicenter	Complications, outcome, mortality	30	30
Oral (5)	Retrospective cohort study, single center	Complications	36	38
Kaliaperumal et al. (12)	Prospective non-randomized trial, single center	Outcome	25	25
Bellut et al. (14)	Retrospective cohort study, single center	Complications	48	65

### Recurrence

**Figure 3:**
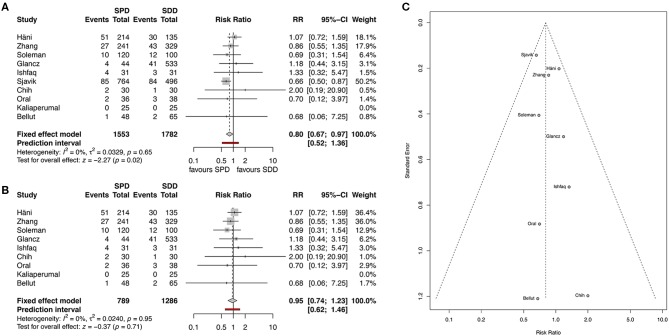
**(A)** Forest plot of recurrence rates. **(B)** Forest plot of recurrence rate after applying the “leave-one-out” method. **(C)** Funnel plot showing homogenous distribution for recurrence. RR, risk ratio; CI, confidence interval; SPD, subperiosteal drain; SDD, subdural drain.

Recurrence rate was reported in all studies. Overall pooled results showed that the use of SPD has a significantly lower recurrence rate than the use of SDD (11.9 and 12.3%, respectively, RR 0.8, 95% CI 0.67–0.97, *I*^2^ = 0%, z = −2.27, *p* = 0.02, Figure 3A). After applying the “leave-one-out” method, Sjavik et al. (13) was identified as a single influential study. Therefore, the pooled analysis was repeated without Sjavik et al. showing no significant difference in recurrence rates between the groups (12.8 and 10.5%, RR 0.95, 95% CI 0.74–1.23, *I*^2^ = 0%, z = −0.37, *p* = 0.71, Figure 3B). The distribution of the studies in the funnel plot was homogenous and therefore publication bias was not suspected (Figure 3C).

### Morbidity

**Figure 4:**
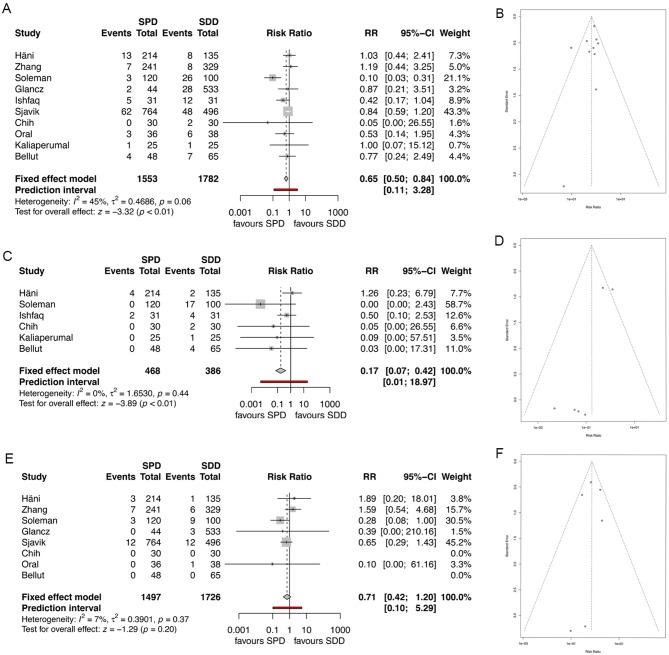
**(A)** Forest plot of morbidity rates. **(B)** Funnel plot showing homogenous distribution for morbidity. **(C)** Forest plot of drain misplacement rates. **(D)** Funnel plot showing homogenous distribution for drain misplacement rates. **(E)** Forest plot of infection rates. **(F)** Funnel plot showing homogenous distribution for infection rates. RR, risk ratio; CI, confidence interval; SPD, subperiosteal drain; SDD, subdural drain.

Overall morbidity was described in all included studies. Overall pooled morbidity rate was significantly lower in the SPD group compared to the SDD group (6.4 and 8.2%, respectively, RR 0.65, 95% CI 0.5–84, *I*^2^ = 44.5%, z = −3.32, *p* = 0.0009; Figure 4A). The corresponding funnel plot showed a homogenous distribution (Figure 4B).

Six studies specifically analyzed drain misplacement rate or parenchymal injury rate due to drain misplacement (4, 7, 8, 10, 12, 14). Overall misplacement rate and consecutive parenchymal injury rate was significantly lower in the SPD group compared to the SDD group (1.2 and 7.8%, respectively, RR 0.17, 95% CI 0.07–0.42, *I*^2^ = 0%, z = −3.4, *p* = 0.0001, Figure 4C), while the funnel plot showed homogenous distribution of the studies (Figure 4D).

Eight studies described infection rates (4, 5, 7–9, 11, 14, 19). Overall pooled infection rate was lower in the SPD group, while significance was not seen (1.7% for SPD and 1.9% for SDD; RR 0.71, 95% CI (0.42–1.25), *I*^2^ = 7.3%, z = −1.29, *p* = 0.20, Figure 4E). The distribution of the studies was homogenous (Figure 4F).

### Mortality

**Figure 5:**
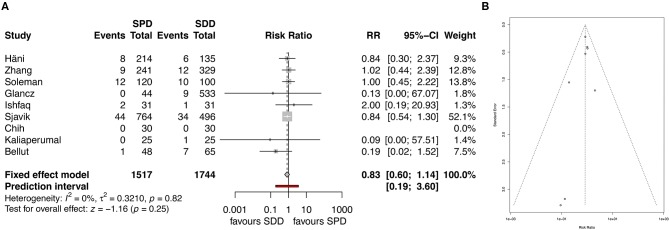
**(A)** Forest plot of mortality rates. **(B)** Funnel plot showing homogenous distribution for mortality rates. RR, risk ratio; CI, confidence interval; SPD, subperiosteal drain; SDD, subdural drain.

All studies, except one, reported on mortality rates (4, 7–14). Overall mortality was lower in the SDD group, without showing significance (5.0% for SPD and 4.6% for SDD; RR 0.83, 95% CI 0.6–1.14, *I*^2^ = 0%, z = −1.2, *p* = 0.25, Figure 5A). The corresponding funnel plot showed homogenous distribution of studies (Figure 5B).

### Clinical Outcome

**Figure 6:**
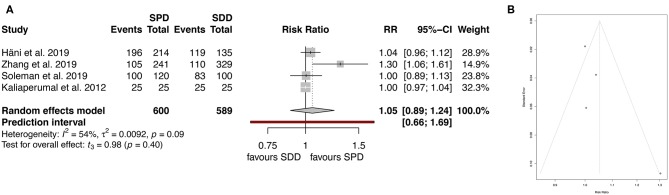
**(A)** Forest plot of favorable clinical outcome (mRS). **(B)** Funnel plot showing homogenous distribution for clinical outcome (mRS). RR, risk ratio; CI, confidence interval; SPD, subperiosteal drain; SDD, subdural drain.

Clinical outcome was assessed by six of the studies included (7–9, 11, 12, 14, 19). After analyzing favorable outcome using mRS the heterogeneity (*I*^2^) was 54%, therefore the random-effect analysis was applied, showing similar rates of good clinical outcome between the groups (89.6% for SPD and 88.9% for SDD; RR 1.05, 95% CI (0.90–1.24), *I*^2^ = 54.2%, *t* = 0.98, *p* = 0.4; Figure 6A). The corresponding funnel plots showed no bias of publication (Figure 6B).

**Figure 7:**
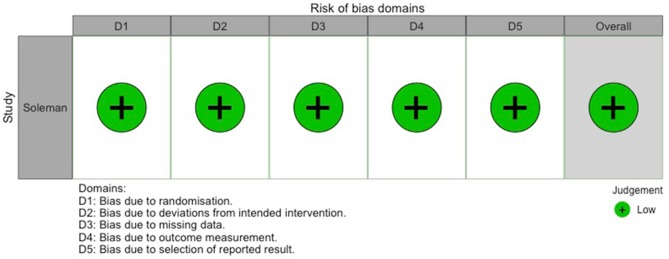
Traffic-light-plot depicting risk of bias for the randomized-controlled trial by Soleman et al. (7).

### Qualitative Assessment of Studies

Out of the 0 studies included, only Soleman et al. (7) conducted a randomized-controlled trial (RCT). Risk of bias for this study is shown in **Figure 7**. Table 2 contains the quality assessment of all the other studies included.

**Table 2:** Quality assessment of the retrospective cohort studies using the Newcastle Ottawa Scale (NOS) and of the prospective non-randomized cohort studies using Robins-1.

**References**	**NOS**	**Robins-1**
Häni et al. (8)	8	–
Zhang et al. (9)	9	–
Glancz et al. (11)	7	–
Ishfaq (10)	–	Intermediate
Sjavik et al. (13)	8	–
Chih et al. (4)	–	Intermediate
Oral (5)	6	–
Kaliaperumal et al. (12)	–	Low-intermediate
Bellut et al. (14)	8	–

“*–,” not applicable*.

## Discussion

The aim of this meta-analysis was, to evaluate whether there is a difference in recurrence rate of cSDH following insertion of SPD or SDD. This meta-analysis shows that SPD has a significantly lower risk for recurrence, drain misplacement and intraparenchymal injury, as well as morbidity, when compared to SDD. Concerning mortality and clinical outcome, no significant difference was seen between the drain groups.

Although cSDH remains one of the most common neurosurgical disease entities, there is a paucity of studies on outcome differences in cSDH after insertion of SPD and SDD. Recurrence in cSDH occurs in ~10% of surgically drained patients and causes higher morbidity in the affected patients (20, 21). It was shown, that the insertion of a drain after burr-hole drainage of cSDH reduces recurrence rates and improves outcome significantly (3). Technical nuances, amongst others, the insertion of a drain, or the localization of the drain (above or under the bone), seem important to achieve better surgical outcome. For this reason, in recent years, some authors compared the outcome of SPD insertion with SDD insertion after burr-hole drainage of cSDH. Unfortunately, to date, only one RCT exists on the matter, while all other series are of retrospective nature, *post-hoc* analysis of prospective cohorts, or consist of rather small cohorts.

To date, the only RCT published comparing SPD and SDD showed no significant difference in recurrence rate between the two groups (11.9 and 12.3%, respectively) (7). Similarly, Zhang et al. (11 and 13%) (9), Chih et al. (7 and 10%) (4), Sjavik et al. (11 and 16%) (13) and Oral et al. (6 and 8%) (5) did not show a significant difference in recurrence rates, while the study by Kaliaperumal et al. showed no recurrences at all (12). Häni et al. (24 and 22%), Glancz et al. (9 and 8%) (11) and Ishfaq (13 and 10%) (10) showed lower recurrence rates in the SDD group compared to the SPD group; however, statistical significance was not observed in any of these studies.

Sjavik et al. (13) compared in their study three different drain techniques: (1) SDD with irrigation, (2) passive SDD, (3) active SPD. For our analyses, both SDD types were included in the SDD group. In Sjavik et al.'s analysis passive SDD showed a higher recurrence rate compared to active SPD. However, the drains with subdural irrigation showed no significant difference in recurrence rate; hence, their results could be due to the active negative pressure within the drain and is most probably less affected by the drain's location. Data on the influence of inserting an active suction drain after surgical drainage of cSDH is sparse, while to our knowledge trials comparing the outcome of active vs. passive drainage do not exist.

Parenchymal hemorrhage due to drain misplacement is a feared complication when inserting an SDD after burr hole drainage of cSDH, potentially increasing perioperative morbidity and mortality (2, 7, 22). All studies reporting misplacement rates showed lower rates in the SPD group (4, 7, 8, 10, 12, 14). The overall misplacement rates in the included studies was 1.2% for SPD and 7.8% for SDD, while only the study by Häni et al. showed a higher misplacement rate in the SPD group. In their *post-hoc* analysis, Häni et al. distinguished between two groups, defined by the year of treatment within their study, namely: “SDD recommended” (*n* = 214, with a possibility to switch to SPD) and SDD treated (*n* = 135). All misplacements (*n* = 6) occurred in the “SDD recommended” group, however, four of these patients ultimately were treated with an SPD since the placement of an SDD was difficult and caused brain injury. These patients were however allocated to the SPD group leading to the above-described overall misplacement rate of 1.2% in the SPD group.

Based on the included studies mortality rate in the SPD group was 5% and in the SDD group 4.6%. Interestingly, although drain misplacement, parenchymal injury, and overall morbidity rate were significantly higher in the SDD group, mortality rate was comparable in both drain groups. This might be because the studies included were not sufficiently powered to show such an association. Further, most intraparenchymal injuries might lead to transient or permanent morbidity (e.g., hemiparesis or aphasia), however, they are not necessarily fatal.

Our analysis showed no significant difference between the groups concerning infection rate, while SPD showed significant lower rates of overall morbidity. The infection rates in both groups, based on the included studies, were 1.7% for SPD and 1.9% for SDD. The only study showing significantly lower rates of infections in the SPD group (2 vs. 9%) was the RCT by Soleman et al. (7). A possible explanation is that other studies (5, 11), which also showed absolutely lower numbers of infection in the SPD group, were underpowered for such an analysis and therefore statistical significance was not reached (5, 11). Infection rate in cSDH are reported to occur in up to 20% of the cases, while empyema rates are much lower (around 2%) (6). In theory, SPD might lower the risk of subdural empyema or other types of “deep” infections, since no foreign material is placed within the subdural cavity or in proximity to the cortical surface.

Santarius et al. showed that the insertion of a drain improves outcome and lowers mortality in cSDH when compared to no drain insertion (3). Based on our pooled analysis the type of drain did not seem to influence clinical outcome. From the included studies, only Kaliaperumal et al. showed significantly better outcome in the SPD group compared to the SDD group (12). All other studies describing outcome showed comparable clinical outcomes in both groups. Interestingly, although recurrence rates, overall morbidity, and intraparenchymal injury rates were significantly lower in the SPD group, clinical outcome was comparable between the groups. One possible explanation might be that the included studies were not powered to detect the true outcome within the groups. Further, morbidity due to misplacement of the drain might be only transient showing an improvement with time. Last, in most studies clinical outcome was assessed in a retrospective manner, with many dropouts or missing information, potentially leading to biased results. Studies focusing and analyzing the outcome after insertion of an SPD compared to SDD in a prospective manner are still needed.

Recently published analysis by Pranata et al., Xie et al., and Ding et al. confirmed our results (23–25). Xie et al. (24), Ding et al. (25), and Pranata et al. (23) also observed significantly lower recurrence rates for SPD as opposed to SDD (Xie et al. and Ding et al.: 12 and 12.7%, Pranata et al.: 12 and 13.2%).

Significantly lower misplacement rates were reported in favor of SPD by Xie et al. (1.% and 2.6%) (24), Ding et al. (0.6 and 2.3%) (25) and Pranata et al. (2.2 and 4.7%) (23). Mortality rates were similar for both groups in all three analysis. Xie et al. (24) reported a mortality rate of 3.7 and 3.8%, in favor of SPD. Ding et al. (25) observed a lower mortality rate for SDD (4.8 and 4.5%) while Pranata et al. (23) published much higher absolute values of 15.7 and 9.4% in favor of SDD.

Comparable outcome rates for both groups were published by Xie et al. (87.4 and 82.1%) (24) Ding et al. (68.1 and 67.5%) (25) and Pranata et al. (percentage not available) (23).

Our assessment and classification of the included studies differed from the meta-analyses by Pranata et al. and Ding et al. while concurred with Pranata et al., Xie et al., Ding et al. (23–25). We considered the study by Soleman et al. (7) the only RCT amongst the included studies, while Pranata et al. and Ding et al. (23, 25) considered Häni et al. (8) and Kaliaperumal et al., respectively, only Kaliaperumal et al. (12), as additional RCTs. We did not consider the study by Kaliaperumal et al. an RCT since the drain type was not randomized (the patients were assigned alternately to SPD and SDD). Similarly, the study by Häni and colleagues was not considered by us as an RCT, since it was not primarily designed to compare the recurrence rate after insertion of SPD or SDD, but rather a *post-hoc* analysis of their RCT initially designed to assess the need for follow up CT after evacuation of cSDH (8). In addition, as opposed to Pranata et al., Xie et al., and Ding et al. (23–25), we combined the two different subdural drain types described by Sjavik et al. into one SDD group (13) and we included the “as treated” rather than the “intention to treat” results from the study by Häni et al. (8) in our analysis. Ding et al. was the only study to report subgroup analysis in their analysis (25). Due these reasons, some of our results differed from the previous reports published.

## Limitations Of the Study

Despite conducting a current systematic review and meta-analysis of the existing literature, our study consists of some limitations. First, we only searched two databases (Pubmed and Embase) and only included English literature, which carries a risk of omitting important data published elsewhere. Second, in our review and analysis we included RCTs, as well as non-randomized prospective cohort studies and retrospective cohort studies. Therefore, the data included was somewhat heterogeneous, potentially influencing the validity of our results. However, to date only one RCT and very few well-designed prospective trials have been published on the matter. Third, even though we assessed for publication bias we cannot exclude a general publication bias, due to unpublished negative studies, which are not included in our meta-analysis. Last, most data included in this meta-analysis derived from retrospective cohorts, inherent to all limitations of such studies, potentially influencing the validity of the results as well.

## Conclusion

Based on the existing evidence, the insertion of an SPD after burr-hole drainage of cSDH seems superior to SDD, in terms of recurrence, overall morbidity, drain misplacement, and intraparenchymal injury rates, while showing comparable infection rates, mortality, and clinical outcome. Therefore, the insertion of SPD after drainage of cSDH should be encouraged. Further prospective studies on the clinical outcome and mortality after insertion of SPD or SDD should be focus of future research.

## Data Availability Statement

All datasets generated for this study are included in the article/supplementary material. The R code is available upon request.

## Author Contributions

LG carried out the database search, review of the literature, data extraction, and analysis as well as drafting of the manuscript. NH was the second researcher who independently reviewed the literature and performed quality and bias assessments. JS the senior author, overviewed the project and took final decisions about inclusion of papers if LG and NH could not reach a conclusion. He critically reviewed the manuscript.

## Conflict of Interest

The authors declare that the research was conducted in the absence of any commercial or financial relationships that could be construed as a potential conflict of interest.
